# The effect of the combined use of silver diamine fluoride and potassium iodide in disrupting the plaque biofilm microbiome and alleviating tooth discoloration: A systematic review

**DOI:** 10.1371/journal.pone.0252734

**Published:** 2021-06-11

**Authors:** Anahita Haiat, Hien Chi Ngo, Lakshman Perera Samaranayake, Kausar Sadia Fakhruddin

**Affiliations:** 1 UWA Dental School, The University of Western Australia, Perth, WA, Australia; 2 Faculty of Dentistry, University of Hong Kong, Hong Kong, Hong Kong; 3 Department of Preventive and Restorative Dentistry, College of Dental Medicine, University of Sharjah, Sharjah, United Arab Emirates; Universitat Bern, SWITZERLAND

## Abstract

Silver diamine fluoride (SDF) is used in minimally invasive dentistry for arresting dental caries. However, discoloration of teeth is a significant side effect that has limited the use of SDF. Hence, the application of potassium iodide (KI) following SDF has been proposed to ameliorate the staining. Although antimicrobial activity is one of the major mechanisms of the caries-arresting effect of SDF, the antimicrobial potency of SDF/KI combination is unclear. Thus, the primary objective of this systematic review was to appraise the studies on the antimicrobial efficacy of SDF/KI combination on cariogenic microbes. The secondary objective was to summarize the evidence on the potential of KI in reducing the discoloration associated with the application of SDF. Electronic databases of Medline via PubMed, Cochrane Library, Web of Science, and EBSCO host were searched for English language manuscripts from January 2005 to 15^th^ November 2020. The reference lists of these manuscripts were manually searched for additional studies. Twelve studies were included in the final analysis, seven of which have investigated the antimicrobial efficacy of SDF/KI, and the rest have examined the anti-staining potential of KI. The exploratory findings from the reviewed articles revealed the promising antimicrobial potential of SDF/KI on cariogenic microbes associated with dentine caries. There is, however, contradictory evidence on the effect of SDF/KI on tooth color. The reviewed *in-vitro* studies indicated significant effectiveness of KI in preventing staining. A clinical trial on primary dentition showed 25% reduction in the incidence of staining by SDF after applying KI, while a clinical study on root caries in adults showed no significant effect. Within the methodological limitations of this review, we conclude that for arresting dental caries, SDF could be combined with KI, as there may be a lower likelihood of staining. Further, well-designed clinical trials on the antimicrobial and anti-staining effect of SDF/KI are needed to obtain more robust evidence.

## Introduction

The management philosophy of dental caries has gone through an evolutionary reckoning over the last century. Traditionally this process entailed a purely surgical “drill and fill” approach, with the G.V. Black cavity design and the “extension for prevention” paradigms as its cornerstone [1]. However, an improved understanding of the pathophysiological processes of dental caries in recent years has led to the emergence of the *Minimal Intervention Dentistry (MID)*, which emphasizes the critical importance of preserving the integrity of the natural tooth structure and adopts a biological approach in the management of caries lesions [2–4]. One such approach has been attributed to Nishino et al. [5], who described the caries-arresting properties of silver diamine fluoride (SDF) in 1969. Since then, numerous randomized controlled trials (RCT) and systematic reviews have established that SDF is effective in arresting dental caries, particularly in the primary dentition [6–19] as well as in root caries lesions of the permanent dentition [20–25].

A primary mechanism of the caries-arresting effect of SDF is its microbicidal effect on cariogenic biofilm [26, 27]. Laboratory studies have revealed the strong inhibitory effect of SDF on monospecies biofims of *Sreptococcus mutans*, *Actinomyces naeslundii*, *Sreptococcus sobrinus* and *Lactobacillus acidophilus* [28, 29]. One study also showed that SDF inhibited the development of a multispecies biofilm of *S*. *mutans*, *S*. *sobrinus*, *Lactobacillus rhamnosus*, *L*. *acidophilus*, and *A*. *naeslundii* on SDF-treated dentine samples [30].

The antimicrobial activity of SDF is mainly attributed to its elemental silver content. Historically, silver has been used as an antimicrobial compound in the treatment of ulcers [31]. Today, it is still used in the treatment of burn wounds, preventative eye care, and on medical implant surfaces to minimize biofilm growth [32]. There are several ways in which silver exerts its antimicrobial effect. First, it interacts with sulfhydryl groups on the surface of microorganisms by replacing the hydrogen atoms, which leads to the formation of an S–Ag bond and blocks cellular respiration and electron transfer [32–35]. It has been noted that, in fungi such as *Candida* species, silver ions bind to vital functional groups of cytoplasmic or membrane-bound enzymes, rendering them inactive [36]. In bacteria such as *Pseudomonas aeruginosa*, they inhibit cell division and damage cell envelope and cellular contents [37]. Additionally, silver ions also interact with nucleic acid bases, resulting in DNA perturbations [38]. However, it must be noted that the foregoing antimicrobial activity of silver ions, all described in planktonic phase microbial suspensions, may not directly extrapolate into their biofilm phase counterparts, due to the extracellular matrix of the biofilms that may act as a physical barrier [39].

Despite such a convincing database on the antimicrobial efficacy of SDF in arresting dental caries, the clinical application of SDF has been rather limited to date. Although this may partly be due to the fact that SDF has not been formally licensed for the management of dental caries in many jurisdictions [40], the main barrier appears to be the discoloration of arrested carious lesions associated with the application of SDF [41], which limits both the patient’s and parents’ acceptability of its use [41]. It is well known that parents have a low tolerance of SDF staining, particularly on anterior teeth due to obvious aesthetic reasons [42].

The staining that follows application of SDF is due to the precipitation of silver phosphate (Ag_3_PO_4_) [42, 43]. To overcome this issue and expand the clinical use of SDF, Ngo et al. have proposed a method to overcome the staining due to SDF [44]. This entails the application of a layer of potassium iodide (KI) over the initial layer of SDF, which then reacts with the free silver ions in SDF and prevents the formation of silver phosphate precipitate. Since then, several studies [45–47] have reported that the application of KI following SDF forms a yellow silver iodide precipitate and prevents black staining of teeth. Detsomboonrat et al. [48] observed a significant immediate reduction in staining due to SDF following application of KI in a dose-dependent manner.

Although iodine solutions have been used in the past as an antiseptic for managing wounds and wound infections [49], and iodine potassium iodide (IKI) is commonly used in dentistry as an endodontic irrigant [50, 51], the antimicrobial potency of iodide ions (I^−^) in aqueous potassium iodide (KI) is under debate [52]. On the other hand, Zhao et al. [47] have suggested that KI may reduce the antimicrobial efficacy of SDF.

To the best of our knowledge, no systematic review or meta-analysis on the antimicrobial efficacy of SDF/KI combination against cariogenic microbes has been conducted previously. Thus, the primary objective of this present study was to systematically review the *in-vitro* and *in-vivo* evidence on the antimicrobial efficacy of SDF/KI on cariogenic microbes. Furthermore, several studies have demonstrated the aesthetic advantage of potassium iodide in preventing stains that ensue SDF treatment. Hence, our secondary objective was to summarize and appraise the available evidence on the efficacy of potassium iodide in reduction or prevention of the staining associated with SDF.

## Material and methods

### Data sources

Two investigators (AH and KSF) performed an electronic search of Medline via PubMed, Cochrane Library, Web of Science, and EBSCO host databases for English language manuscripts published between January 2005 and 15^th^ November 2020. Twelve studies were selected, seven of which have investigated the antimicrobial efficacy of SDF/KI, and five studies examined the anti-staining potential of KI following SDF application.

Two specific review questions were formulated using the PICO framework as follows:

1. Does SDF/KI (I) have an effective antimicrobial action (O) compared to distilled water/saline/chlorhexidine (C) against cariogenic microorganisms (P)?
2. Does the post-SDF application of KI (I) reduce or prevent staining (O) compared to water/no KI application (C) in carious lesions in primary and permanent dentition (P)?

### Search terms

A particular search string was structured for each of the databases, which included the following search terms:

Silver diamine fluoride AND (caries OR carious lesion OR biofilm) AND (dentin OR dentine OR dentinal); potassium iodide AND (caries OR carious lesion OR biofilm) AND (dentin OR dentine OR dentinal); silver diamine fluoride AND (potassium iodide OR KI) AND (staining OR discoloration); silver diamine fluoride AND (antimicrobial OR antibacterial OR antifungal OR bacteriostatic OR bactericidal).

### Inclusion criteria

#### SDF/KI antimicrobial effect

English language articles, dentine biofilm, *in-vitro*, *in-vivo*, *ex-vivo*, *in situ* oral-biofilm model, asymptomatic dentine/dentinal caries, human dentine/dentin block of primary/permanent teeth, cariogenic microbe/bacteria, cariogenic yeasts/Candida/Candida species, SDF/SDF-KI antimicrobial effect (caries arrest), SDF/SDF-KI pre-post bacterial reduction (caries arrest), SDF/SDF-KI pre-post cariogenic, yeast/Candida/Candida species reduction (caries arrest)

#### KI anti-staining potential

English language articles, *in-vivo*/*in-vitro* study design, human dentine/dentin blocks, dentine/dentinal caries lesion, post-SDF application of KI and stain reduction, post-SDF application of KI and stain reduction, qualitative/quantitative assessment of silver stain reduction

### Exclusion criteria

#### SDF/KI antimicrobial effect

Incomplete outcome data, bovine dentine, enamel caries, child or adult patient on an antibiotic, conference/poster presentation, abstracts, case reports, unpublished information/grey literature, opinion articles, scoping reviews, systematic reviews, umbrella reviews

#### KI anti-staining potential

Incomplete outcome data, bovine dentine, enamel, conference/poster presentation, abstracts, case reports, unpublished information/grey literature, opinion articles, scoping reviews, systematic reviews, umbrella reviews

### Outcome

The combined antimicrobial effect of SDF/KI on dentine biofilm and the anti-staining potential of KI, post-SDF application on dentine caries.

### Electronic data search and analysis

The Preferred Reporting Items for Systematic Reviews and Meta-Analyses (PRISMA) guidelines [53, 54] were adopted for this study. A total of 563 papers were identified through a search of the electronic databases, after which 327 articles were removed, leading to 236 studies. Overall, 15 articles were selected for full-text review. After reviewing the articles, 12 studies were deemed suitable for the current systematic review (S1 Fig). Following full text assessment, the characteristics of each study were recorded using the Cochrane pattern [55] to determine the study design, setting, specimen preparation, and test-microbes, as presented in Tables 1 and 2.

**Table 1:** Characteristics of the included studies on the antimicrobial effect of SDF/KI.

Study	Setting *(dentinal/root caries lesion in primary/ permanent teeth)*	Setting *(human dentine blocks OR biofilm model)*	Inclusion and exclusion criteria	Antimicrobial agents	Test Microbes	No. of primary /permanent teeth or dentine blocks or biofilm samples	Test & control group	Timepoint
*In vivo* -antimicrobial efficacy								
**Karched et al., 2019**	**X**	**-**	**X**	• 38% SDF • 38%SDF/KI • 2% CHX • Sterile saline	*Biofilm isolated from carious lesions (Streptococcus mutans and anaerobes)*	25 permanent teeth	**X**	Evaluation after 48 hours of incubation
*In vitro* -Antimicrobial efficacy								
**Abdullah et al., 2020**	-	**X**	**X**	• 38% SDF • 31.3% SDF • KI • 31.3% SDF/KI • Sterile distilled water • 2% CHX	*In-situ oral biofilm*	90 biofilm samples	**X**	In-situ biofilms were developed intraorally and collected after 6 hours for evaluation
**Vinson et al., 2018**	-	**X**	-	• 38% SDF • KI • 38% SDF/KI	*Streptococcus mutans*	Unspecified	**X**	Evaluation after 24 hours
**Hamama et al., 2015**	**-**	**X**	**X**	• 38% SDF/KI • 2% CHX • 2% CHX + 38% SDF/KI • Carisolv • Carisolv + 38% SDF/KI • Papacarie • Papacarie + 38% SDF/KI	*Streptococcus mutans*	45 dentine blocks	**-**	Evaluation after 5 minutes of dentine disc treatment
**Knight et al., 2009**	**-**	**X**	**X**	• SDF/KI • KI • SDF	*Streptococcus mutans*	40 dentine blocks	**X**	Evaluation after 14 days
**Knight et al., 2007**	**-**	**X**	**X**	• SDF/KI	*Streptococcus mutans*	35 dentine blocks	**X**	Evaluation after 14 days
**Knight et al., 2005**	**-**	**X**	**X**	• SDF • SDF/KI • KI	*Streptococcus mutans*	40 dentine blocks	**X**	Evaluation after 14 days

X = mentioned/present in the included publication;— = not mentioned, in the included publication.

**Table 2:** Characteristics of the included studies on the anti-staining effect of KI post-SDF application.

Study	Setting *(dentinal/root caries lesion in primary/ permanent teeth)*	Setting *(human dentine blocks)*	Inclusion and exclusion criteria	Anti-staining agent (potassium Iodide)	No. of primary /permanent teeth/ dentine blocks	Test & control group	Timepoint
*In vivo* -KI anti-staining effect							
**Turton et al., 2020**	**X**	**-**	**X**	• 38% SDF • 38% SDF/KI • 38% AgF • 38% AgF/KI	2335 carious lesions (primary teeth)	**X**	Evaluation after 6 months
**Li et al., 2016**	**X**	**-**	**X**	• Soda water • 38% SDF • 38% SDF/KI	100 root caries lesions (permanent teeth)	**X**	Evaluation after 30 months
*In vitro* -KI anti-staining effect							
**Zhao et al., 2019**	**-**	**X**	**-**	• 38% SDF/KI • 38% SDF • Water	60 dentine blocks	**X**	Evaluation after 24 hours
**Patel et al., 2018**	**X**	**-**	**-**	• 38% SDF • 38% SDF/KI	20 carious primary molars	**X**	Evaluation at regular intervals for a period of 7 days
**Nguyen et al., 2017**	**X** (both carious teeth and teeth with minimal decay)	**-**	**-**	• 38% SDF/KI + class I composite restoration (caries-free) • 38% SDF + class I composite restoration (caries-free) • 38% SDF/KI (existing caries) • 38% SDF (existing caries) • 38% SDF/KI (caries-free) • 38% SDF (caries-free) • 38% SDF/KI + class I RMGIC restoration (caries-free) • 38% SDF + class I RMGIC restoration (caries-free) • 38% SDF/KI + class I GI restoration(caries-free) • 38% SDF + class I GI restoration (caries-free)	45 molars	**X**	Evaluation after 4 weeks

X = mentioned/present, in the included publication;— = not mentioned, in the included publication.

To answer the clinically relevant question, a three-step approach of evidence-based analysis was employed. In stage one, two reviewers (AH and KSF) independently screened the studies’ titles and abstracts using the defined inclusion and exclusion criteria. In cases where it was challenging to gather sufficient information from an abstract, the full text was assessed for study validity and clinical importance. In the second stage, appraisal and selection of articles were performed by AH and KSF. In cases where there was disagreement between the reviewers, a third reviewer (HCN) was consulted. Finally, another reviewer (LPS) reassessed and validated the articles to be included in the review. A spreadsheet method was employed to extract information from the included studies and ensure that the eligibility criteria were met. At this stage, the studies’ reference lists were manually searched to identify potential additional studies that could be considered. The identified articles were compiled using a bibliographic software tool, Endnote version 9 (Clarivate Analytics, USA).

### Quality assessment and overall risk of bias

In the third stage, the Cochrane Collaboration risk of the bias assessment tool [55] was used by two reviewers (AH and KSF) to assess the methodological quality of the studies. According to the evidence pyramid, *in-vitro* studies have the lowest level of evidence due to the possible risk of “false-positive” results, lack of external validity, and generalizability to real clinical situations [56]. However, to date, there has been no validated guidelines or checklists for reporting *in-vitro* studies [57]. Since the methodological framework of clinical research and *in-vitro* studies is somewhat similar, taking leads from the consolidated standards of reporting trials (CONSORT), we checked the transparency and quality of included *in-vitro* trials [58]. Items such as sample size, specimen preparation and handling, allocation sequence, randomization, and blinding were assessed for inclusion in this systematic review. Accordingly, as illustrated in Table 3, papers were classified as low, unclear, and high-risk, with the consensus being reached through discussion with the third reviewer (HCN) in cases of discrepancy. Owing to the heterogeneity in the study design and outcome of the selected studies, no meta-analysis was performed.

**Table 3:** Risk of bias of the included studies.

Study	Selection bias	Selection bias	Selection bias	Performance bias	Detection bias	Reporting bias	Confounding bias
	Baseline characteristics similarity/appropriate control selection	Allocation concealment	Randomization	Blinding of Researchers	Blinding of outcome assessors	Selective outcome reporting	Account for confounding variable
Antimicrobial effect of SDF/KI							
**Karched et al., 2019**	**+**	**?**	**?**	**?**	**?**	**+**	**+**
**Abdullah et al., 2020**	**+**	**?**	**?**	**?**	**?**	**+**	**+**
**Vinson et al., 2018**	**?**	**?**	**?**	**?**	**?**	**?**	**?**
**Hamama et al., 2015**	**+**	**?**	**+**	**?**	**?**	**+**	**?**
**Knight et al., 2009**	**+**	**?**	**?**	**?**	**?**	**+**	**?**
**Knight et al., 2007**	**+**	**?**	**?**	**?**	**?**	**+**	**?**
**Knight et al., 2005**	**+**	**?**	**?**	**?**	**?**	**+**	**?**
**Anti-staining effect of KI**							
**Turton et al., 2020**	**+**	**+**	**+**	**?**	**-**	**+**	**?**
**Li et al., 2016**	**?**	**+**	**+**	**+**	**+**	**+**	**?**
**Zhao et al., 2019**	**?**	**?**	**+**	**?**	**?**	**+**	**?**
**Patel et al., 2018**	**?**	**+**	**?**	**?**	**?**	**+**	**?**
**Nguyen et al., 2017**	**-**	**?**	**?**	**?**	**?**	**+**	**?**

Risk of bias legends: + (Low risk of bias);—(High risk of bias);? (Un-clear risk of bias).

## Results

The results of the included studies are summarized in Tables 4 and 5.

**Table 4:** Antimicrobial effect of SDF/KI.

Study	Intervention and Comparator	Outcome	Summary Intervention	Summary Comparator
***In vivo* -antimicrobial efficacy**				
**Karched et al., 2019**	• 38% SDF • 38% SDF/KI • 2% CHX • Sterile saline	• ***Total viable counts—Colony forming units***	**SDF/KI—**Median CFU counts/mg of dentine before and after treatment.	**CHX—**Median CFU counts/mg of dentine before and after treatment.
			Total viable anaerobic counts (2.96×10^5^ to 9.2×10)	Total viable anaerobic counts (1.11×10^5^ to 4.81×10^2^)
			Total viable *streptococcus mutans* counts (6.78×10^2^ to 0)	Total viable *streptococcus mutans* counts (1.66×10^3^ to 1.16×10^2^)
			**SDF—**Median CFU counts/mg of dentine before and after treatment.	**Saline—**Median CFU counts/mg of dentine before and after treatment.
			Total viable anaerobic counts (9×10^5^ to 1.67×10^2^)	Total viable anaerobic counts (3.68×10^5^ to 3.31×10^5^)
			Total viable *streptococcus mutans* counts (3.5×10^3^ to 0)	Total viable *streptococcus mutans* counts (5.71×10^2^ to 6.5×10^2^)
***In vitro* -Antimicrobial efficacy**				
**Abdullah et al. 2020**	• 38% SDF • 31.3% SDF • KI • 31.3% SDF/KI • sterile water (-ve control) • CHX (+ve control)	• ***Viability of residual biofilm—real-time PCR with PMA***	Percentage of residual viable bacteria:	
			**38% SDF** (30.64 ± 8.95%)	
			**31.3% SDF** (31.75 ± 17.67%)	
			**31.3% SDF/KI** (38.19 ±13.62%)	
			**2% CHX** (39.55 ±14.18%)	
			• KI alone exerted no antibacterial effect on plaque biofilms (P>0.05) • Non-significant difference in antibacterial efficacy of 38% SDF, 31.3% SDF and 31.3% SDF/KI	
**Vinson et al. 2018**	• 38% SDF • KI • 38% SDF/KI	• ***Microbial Kinetics—Colony forming units***	**SDF—**seven-fold log10 reduction in CFU per mL	
			**KI—**two-fold log10 reduction in CFU per mL	
			**SDF/KI—**four-fold log10 reduction in CFU per mL	
**Hamama et al., 2015**	• 38% SDF/KI • 2% CHX • 2% CHX + 38% SDF/KI • Carisolv • Carisolv + 38% SDF/KI • Papacarie • Papacarie + 38% SDF/KI	• ***Mean percentages of dead and live bacteria—Confocal laser scanning microscopy***	Percentage of viable bacteria:	Percentage of viable bacteria:
			**SDF/KI** (43.9 ±13.0%)	**Carisolv** (99.6± 0.7%)
			**Carisolv + SDF/KI** (72.3±13.5%)	**Papacarie** (80.5± 6.6%)
			**Papacarie + SDF/KI**	**CHX** (81± 10.8%)
			(63.1 ±4.4%)	
			**CHX + SDF/KI** (57.2±21.6%)	
**Knight et al., 2009**	• SDF/KI • KI • SDF	• ***Surface Biofilm -Scanning electron microscopy***	• *S*. *mutans* biofilm covered the surface of disks treated with KI • No biofilm on disks treated with either SDF or SDF/KI	• *S*. *mutans* biofilm covered the surface of control disks
**Knight et al., 2007**	• SDF/KI	• ***Optical Density -Scanning electron microscopy***	Graph depicting the mean values and standard deviations of the optical density readings in the nutrient broth of test and control groups	
**Knight et al., 2005**	• SDF • SDF/KI • KI	• ***Optical Density -Spectrophotometer***	**SDF/KI group** *(Optical density = 0*.*185±0*.*017)*	***Control group*** *(Optical density = 0*.*990±0*.*778)*
			**SDF group** *(Optical density = 0*.*387±0*.*546)*	
			**KI group** *(Optical density = 0*.*590±0*.*641)*	

**Table 5:** Anti-staining effect of KI post-SDF application.

Study	Intervention and Comparator	Outcome	Summary Intervention	Summary Comparator
***In vivo* -KI anti-staining effect**				
**Turton et al., 2020**	• 38% SDF • 38% SDF/KI • 38% AgF • 38% AgF/KI	*• Color scale categories ‘yellow’*, *‘light brown*,*’ ‘dark brown*,*’ ‘black’—Visual assessment*	**SDF/KI—**34.3% lesion darkening	**SDF—**49.1% lesion darkening
			**AgF/KI—**31% lesion darkening	**AgF—**46.1% lesion darkening
**Li et al., 2016**	• Soda water • 38% SDF 38% • SDF/KI	*• Color scale categories ‘yellow’*, *‘light brown*,*’ ‘dark brown*,*’ ‘black’—Visual assessment*	**SDF/KI group—**Final color distribution included 62% black and 32% dark brown lesions	**SDF group—**Final color distribution included 69% black and 25% dark brown lesions
***In vitro* -KI anti-staining effect**				
**Zhao et al., 2019**	• 38% SDF/KI • 38% SDF • Water	• *CIELAB color system—Colorim****eter***	**SDF/KI—**Color coordinates	**SDF—**Color coordinates
			L* = 69.8±7.1	L* = 32.1±4.5
			a* = 2.1±0.6	a* = 8.6±1.5
			b* = 10.5±4.8	b* = 3.6±2.5
			ΔE = 14.4	ΔE = 24.6
				**Water—**Color coordinates
				L* = 55.6±4.3
				a* = 4.6±2.3
				b* = 9.6±6.0
				ΔE = N/A
**Patel et al., 2018**	• 38% SDF • 38% SDF/KI	*• Mean grey value—Digital image analysis*	Graph illustrating the changes in mean grey values of teeth treated with SDF/KI for seven days. No statistically significant changes (P = 0.123) compared with baseline values.	Graph illustrating the changes in mean grey values of teeth treated with SDF for seven days.
**Nguyen et al., 2017**	• 38% SDF/KI + class I composite restoration (caries-free) • 38% SDF + class I composite restoration (caries-free) • 38% SDF/KI (existing caries) • 38% SDF (existing caries) • 38% SDF/KI (caries-free) • 38% SDF (caries-free) • 38% SDF/KI + class I RMGIC restoration (caries-free) • -38% SDF + class I RMGIC restoration (caries-free) • -38% SDF/KI + class I GI restoration(caries-free) • -38% SDF + class I GI restoration (caries-free)	*• CIELAB color system—Colorimeter*	**SDF/KI groups—**Lightness values	**SDF groups—**Lightness values
			Existing caries, no restoration (L* = 71.3±14.2)	Existing caries, no restoration (L* = 53.6±6.7)
			Caries-free, no restorations (L* = 93.7±3.4)	Caries-free, no restorations (L* = 51.2±2.3)

### Primary objective

#### In-situ biofilm model in permanent teeth

Abdullah et al. [59] tested the antimicrobial efficacy of SDF, KI, and SDF/KI on *in-situ* biofilms. Five participants were instructed to wear a maxillary intraoral appliance, each having six removable wells for biofilm collection, which were removed after 6 hours. The experiment was repeated three times for each participant, yielding 90 biofilm samples divided into the six test groups of 38% SDF, 31.3% SDF, KI, 31.3% SDF/KI, sterile distilled water, and 2% CHX. They found that KI did not have any antibacterial effect while both SDF and SDF/KI had potent antibacterial efficacy without any statistically significant difference between the two. Thus, they concluded that KI does not modulate the antibacterial efficacy of SDF.

#### Deep dentinal caries lesions in permanent teeth

An *in-vivo* study by Karched et al. [60] evaluated the effect of SDF and SDF/KI on bacteria in deep dentinal lesions. They employed five patients, each having multiple carious lesions for applications of SDF, SDF/KI, CHX, and sterile saline on separate lesions. Dentin samples were taken before and immediately after the intervention and were cultured for microbiological assessment. Their results showed that both SDF and SDF/KI exhibited complete elimination of *S*. *mutans* and 90% reduction of total anaerobic bacteria in laboratory culture without any statistically significant difference between the two groups.

#### In-vitro effect of SDF/KI on cariogenic biofilm

Four *in-vitro* studies [61–64] have examined the action of SDF/KI against *S*. *mutans* on dentin blocks prepared from extracted non-carious human teeth. Hamama et al. [61] utilized *S*. *mutans* isolated from caries lesions. They demonstrated the antimicrobial potency of SDF/KI compared to control groups by counting the live/dead mass ratio of *S*. *mutans* in the dentinal tubules of the dentin blocks.

Contrastingly, the bacterial supply source in experiments by Knight and colleagues [62–64] is not specified. In two separate studies [62, 63], they used a chemostat system that provided constant *S*. *mutans* over the dentin disks placed over vials. Measurements of the optical density of the solution in the vials were taken. A lower optical density in the vials corresponding to dentin disks treated by SDF and SDF/KI compared to control groups was observed. They concluded that SDF and SDF/KI had significantly prevented the penetration of *S*. *mutans* into the dentin disks. In another trial, they examined the outer surfaces of dentin disks with scanning electron microscopy [64]. They found that the application of either SDF or SDF/KI inhibited the growth of *S*. *mutans* biofilm, whereas KI alone did not show this capacity.

Vinson et al. [65] incubated *S*. *mutans* (ATCC) in tissue culture plates and measured the effects of SDF, SDF/KI, and KI on the biofilms by assessing the colony-forming units. Their findings indicated that all three groups of SDF, SDF/KI, and KI significantly reduced the viability of established *S*. *mutans* biofilms. However, SDF alone showed the most significant overall disruption of the biofilm.

### Secondary objective

#### In-vivo application of SDF/KI on carious lesions in primary teeth

Turton et al. [66] compared the efficacy of the application of KI on the reduction of the staining following SDF and silver fluoride (AgF) treatments on carious lesions in primary teeth. A visual color assessment was carried out on 2335 carious lesions at a 6-month recall using a color scale that included ‘yellow’, ‘light brown’, ‘dark brown’, and ‘black’. A lesion was determined to have darkened if it had progressed into a darker category. They estimated that the application of KI reduced the incidence of darkening by approximately 25% with either AgF or SDF application in isolation.

#### In-vivo application of SDF/KI on root caries lesions in elderly

The effects of SDF/KI on arresting dental root caries, as well as on the aesthetic outcomes were investigated by Li et al., using a RCT on an elderly population [67]. The study involved 100 root caries lesions, and the two test groups received either SDF or SDF/KI treatments at baseline and annually until reassessment after 30 months. They reported that the application of KI following SDF reduced the staining immediately, but on the 30-month review, the lesions still developed dark staining. Interestingly, they reported no significant difference in the final color outcomes between the SDF and SDF/KI groups, after 30 months.

#### Dentin blocks of permanent teeth

Zhao et al. [68] investigated the effect of SDF and SDF/KI on the color of dentine slices prepared from non-carious human third molars. The slices were stored in a demineralizing solution to create artificially induced caries, and following the application of SDF or SDF/KI, they were kept in artificial saliva for 24 hours. Color assessments were done using the CIELAB system with a colorimeter. Their findings showed that the samples treated with SDF/KI had a higher `lightness value`than the negative control group. In comparison, the samples treated with SDF had a lower `lightness value`, thus concluding that SDF/KI did not cause any adverse color effects. In contrast, the application of SDF alone lead to significant staining of the dentin blocks.

#### Ex-vivo primary molars

Patel et al. [69] tested the *in-vitro* anti-staining efficacy of KI on extracted carious primary molars. Teeth were photographed at baseline and regular intervals up to seven days after applying SDF or SDF/KI, and mean grey values (MGV) of the carious lesions were calculated. They demonstrated that MGV of lesions that had received KI was not statistically different from the baseline values. In contrast, with SDF application alone, the MGV of the lesions steadily decreased over time, indicating a gradual darkening of the lesion.

#### Ex-vivo molars

Nguyen et al. [45] conducted an *ex-vivo* experiment on extracted human molars, unspecified whether they were from primary or permanent dentition. The samples were divided into ten different test groups, each receiving either SDF or SDF/KI treatment. Only two groups had teeth with existing caries, while the others had minimal decay. Six groups of non-carious teeth received different types of cavity preparations and restorative materials, and two groups received SDF and SDF/KI without any previous modifications. Color assessments were done at baseline and after four weeks with a colorimeter using the CIELAB system. They revealed that all groups with SDF/KI treatment, irrespective of whether they received a restoration or not, had minimal to no changes in the lightness value, resulting in no darkening as opposed to groups treated with SDF.

### Assessment of methodologies

#### Sample size

Having an appropriate sample size calculated based on sound assumptions is essential to ensure a high study power [70, 71]. None of the foregoing *in-vitro* studies in this systematic review mentioned whether a sample size calculation was conducted before the experiment. The RCTs on the anti-staining effect of KI [66, 67], stated that sample size was determined based on objectives other than assessing the anti-staining effect of KI. However, the sample size calculation must take into account all the outcomes measured within a clinical trial. It is unclear whether the sample size in those studies was sufficiently large to capture the clinical difference of application of KI post-SDF on tooth color. The studies by Karched et al. [60] and Abdullah et al. [59] were also limited in their sample size, each having only five participants.

#### Attrition bias

Loss to follow-up can lead to the exclusion of patients from a clinical trial. Evaluation errors can result if data is analyzed without adjustment for the unequal loss of patients from the various treatment groups, as the lost patients may not be representative of the remaining patients in the study [72]. The studies by Li et al. [67] and Turton and al. [66] acknowledged that not all of their initial participants were present at the follow-up evaluation, thus leading to attrition bias. They also did not apply intention-to-treat analysis to avoid the selection bias.

#### Quality of reporting

The outcome of the study in each group should be reported with adequate statistical measures such as effect size and confidence intervals in addition to the *p*-value [70, 73]. The studies investigated in this review reported their results solely in terms of *p-*values, thus creating difficulty in the precise interpretation of their clinical significance. In particular, Li et al. [67] communicated their findings on the effect of KI on the tooth color by presenting only the final color distribution of the arrested lesions in the SDF, and SDF/KI treated groups without giving any statistical measures or any details about the initial color distributions prior to treatment. Also, Turton et al. [66] did not specify what proportion of lesions from each initial color group had progressed to a darker color. Clearly, it is clinically relevant to determine the proportion of initially light-colored carious lesions that progress to a darker color compared to the proportion of discolored lesions that are dark at the beginning of the treatment regimen.

Another limitation pertaining to the reviewed studies relates to the protocol of the application of KI. In a clinical study, the intervention must be replicable [74], meaning a therapeutic regimen should include a complete description of the regimens’ details [75]. However, none of the reviewed studies had described the exact quantity and duration of application of KI, thus undermining the reproducibility of the results.

## Discussion

Numerous clinical studies have shown that SDF is an effective caries-arresting agent [6–25]. The antimicrobial action of SDF against cariogenic biofilm is thought to be one of its primary mechanisms of action. However, the staining that follows the application of SDF has significantly diminished its use in pediatric and adult patients. One of the proposed methods for preventing this adverse side effect of SDF is the application of KI immediately following SDF. The current systematic review is the first, to our knowledge, to critically evaluate the *in-vitro* and *in-vivo* studies on the antimicrobial efficacy of SDF/KI on plaque biofilm, and the effect of KI on prevention of tooth discoloration in primary and permanent dentition.

### Antimicrobial efficacy of SDF/KI

All the *in-vitro* and clinical studies reviewed here clearly demonstrate that both SDF/KI, and SDF have potent anti-cariogenic activity. While it has previously been hypothesized that application of KI may reduce the antimicrobial efficacy of SDF by reducing the concentration of silver ions [47], studies reviewed here generally indicate that there is no significant difference between the antimicrobial actions of SDF in isolation, and SDF/KI combination, indicating that KI does not affect the microbicidal action of SDF. Thus, three studies [59, 62, 64] revealed that KI does not exhibit any antimicrobial activity, while one study [65] showed some degree of antimicrobial action of KI.

The *in-vitro* studies inevitably have shortcomings in their ability to replicate the complex intra-oral ecosystem. Five *in-vitro* studies [61–65] used a mono-species biofilm derived from cultures of *Streptococcus mutans*. Nevertheless, the oral plaque microflora causing dental caries is a complex microbial consortium [76]. While Hamama et al. [61] used a wild type *Streptococcus mutans* from human caries lesions to mitigate this problem, these are likely to exhibit pathogenic attributes that differ from natural, *in-situ* biofilms.

A single *in-vitro* study has thus far used a natural *in-situ* biofilm [59] to evaluate SDF/KI’s anti-cariogenic effect, although the biofilm was collected from caries-free individuals. It is known that biofilms of carious lesions have a different composition compared to healthy sites due to a shift towards acidophilic and aciduric bacterial species [77]. Another important strength of the latter study, as opposed to the previous *in-vitro* studies discussed above, is that the viability count of the biofilm flora was performed using real-time PCR, leading to the quantification of both the cultivable and uncultivable flora [78].

In contrast, the *in-vivo* clinical study by Karched et al. [60] analyzed biofilm collected from carious lesions. However, only routine microbiological culture assessments were used, hence enabling the analysis of cultivable bacteria only. A major shortcoming of the studies of Abdullah et al. [59] and Karched et al. [60] is the small sample size of less than ten individuals in each study. Since the composition of plaque biofilm varies within individuals and between individuals, a larger sample size is required to illuminate the true *in-vivo* antimicrobial effect of SDF/KI. Moreover, novel methods such as next generation sequencing (NGS) [79] could be used to determine the complete microbial profile of the biofilm samples and specifically validate the antimicrobial effect of SDF/KI on cariogenic biofilm.

### The anti-staining potential of KI

In general, the *in-vitro* studies reviewed clearly imply that the application of KI to varying degrees prevented tooth discoloration associated with SDF [45, 69]. In contrast, RCT by Li et al. [67] reported no significant effect of KI in the long term over a period of 30 months, and Turton et al. [66] recorded an absence of darkening in 25% of cases. Several explanations could be offered for these discrepant observations. Firstly, all the *in-vitro* studies evaluated the color of the lesions for periods of less than one month. In contrast, the reassessment of the lesions in the studies by Li et al. [67] and Turton et al. [66] was conducted after 30 months and 6 months, respectively. It has been shown that silver iodide, which is formed upon the application of KI following SDF, is highly photosensitive. As a result, it can dissociate into silver and iodine by exposure to light [68]. Although unlikely, the longer observation time in the clinical studies by Li et al. [67] and Turton et al. [66] compared to the *in-vitro* may have resulted in more significant dissociation of the silver iodide present.

On the other hand, the *in-vitro* studies were also conducted under varying experimental conditions not mimicking the natural oral ecosystem, limiting their generalizability and extrapolation. Two of these studies [45, 69] were performed in a dry environment, while Zhao et al. [68] used an artificial salivary flow system. The samples they used were artificially demineralized, which would be structurally different to naturally occurring carious lesions.

Furthermore, the RCTs [66, 67] also had methodological limitations that cast doubt on the validity of their conclusions. In these studies, the carious lesions were left open after applying SDF/KI and were not restored, making them uncleanable and prone to staining. Moreover, it is generally recognized that several factors could lead to extrinsic staining of the tooth structure, including poor oral hygiene, tannins in the diet, and tobacco smoking [80, 81]. It has also been suggested that certain bacterial metabolic products could lead to staining of the soft deposits of dental plaque [81, 82]. These confounders were either not controlled, or were difficult to control in the studies of Turton et al. [66] and Li et al. [67] and may have affected the outcome. Also, the latter workers appear not to have considered any exclusion criteria for the examined teeth, thus failing to consider intrinsic factors impacting the results such as developmental or acquired defects of enamel and dentin, previous trauma to the tooth and pre-existing restorations or root canal treatment, all of which could have predisposed the teeth to discoloration.

In terms of color assessment of the SDF/KI treated teeth, it is noteworthy that the *in-vitro* study by Li et al. [67] subjectively evaluated the color using shade guides. Although shade matching is used frequently to assess differences in tooth color, this approach has several drawbacks. The range of available colors in a shade guide may not cover the total spectrum of tooth shades [83]. Intra and inter-examiner variations can arise due to external factors such as age, eye fatigue, lighting conditions, and in particular, operator experience [84]. It has been shown that individuals’ shade matching ability improves with training [85]. Also, the clinical reports did not mention whether the examiners were appropriately trained, tested or calibrated for their color assessment acumen.

Additionally, Li et al. [67] only had a single examiner assessing each lesion, which could have resulted in individual judgmental errors leading to poor reproducibility of results. Contrastingly, the *in-vitro* studies by Nguyen et al. [45] and Zhao et al. [68] used a colorimeter to assess the color of the lesions using the CIELAB color system. This method has been shown to produce good precision and reliability of tooth color measurement [86, 87]. On the other hand, Patel et al. [69] used a more accurate digital image analysis software that calculated the MGV for color assessment. Nevertheless, the digital approach, which appears to be the more superior, has not been formally validated for tooth color assessment.

Finally, it should be mentioned that one factor which can affect the antimicrobial efficacy and the aesthetic outcome of SDF/KI treatment is the amount of KI applied. As indicated in a study by Sorkhdini et al. [88], an excess amount of KI can reduce the bioavailability of silver ions, thereby reducing the antimicrobial Efficacy of SDF/KI. In contrast, an insufficient amount of iodide ions can lead to excess silver ions, which can cause staining in turn. However, none of the reviewed studies stated the ratio of KI to SDF used in their experiments. Moreover, at present, the protocol of application of commercially available SDF/KI does not specify the exact amount of KI that needs to be applied following SDF.

## Conclusion

The available *in-vitro* and clinical studies in the present review have indicated the promising antimicrobial potential of SDF/KI against the cariogenic microflora in dentine caries.

There is contradictory evidence on the effect of SDF/KI on tooth color. All the reviewed *in-vitro* studies on both primary and permanent teeth have indicated significant effectiveness of KI in preventing tooth staining. In contrast, a single clinical study on root caries in the elderly suggested no effect whatsoever. A clinical trial on primary dentition demonstrated 25% reduction in staining incidence by SDF after applying KI.

However, it should be noted that there is a scarcity of studies on SDF/KI, and the manuscripts reviewed in this systematic review had numerous methodological limitations, as discussed previously. Further, well-designed clinical trials on the antimicrobial and anti-staining effect of SDF/KI are needed to obtain more robust data and address the gaps in our knowledge, particularly on the optimum therapeutic regimen of SDF/KI application for the management of dental caries.

## Supporting information

S1 Fig PRISMA flow chart of the literature search and study selection. (TIFF)

S1 File PRISMA checklist. (DOC)
