# Supplementary figures and images for: The effect of the combined use of silver diamine fluoride and potassium iodide in disrupting the plaque biofilm microbiome and alleviating tooth discoloration: A systematic review
Source: PLoS One. 2021 Jun 11;16(6):e0252734. doi: 10.1371/journal.pone.0252734 (PMC8195348; doi:10.1371/journal.pone.0252734)

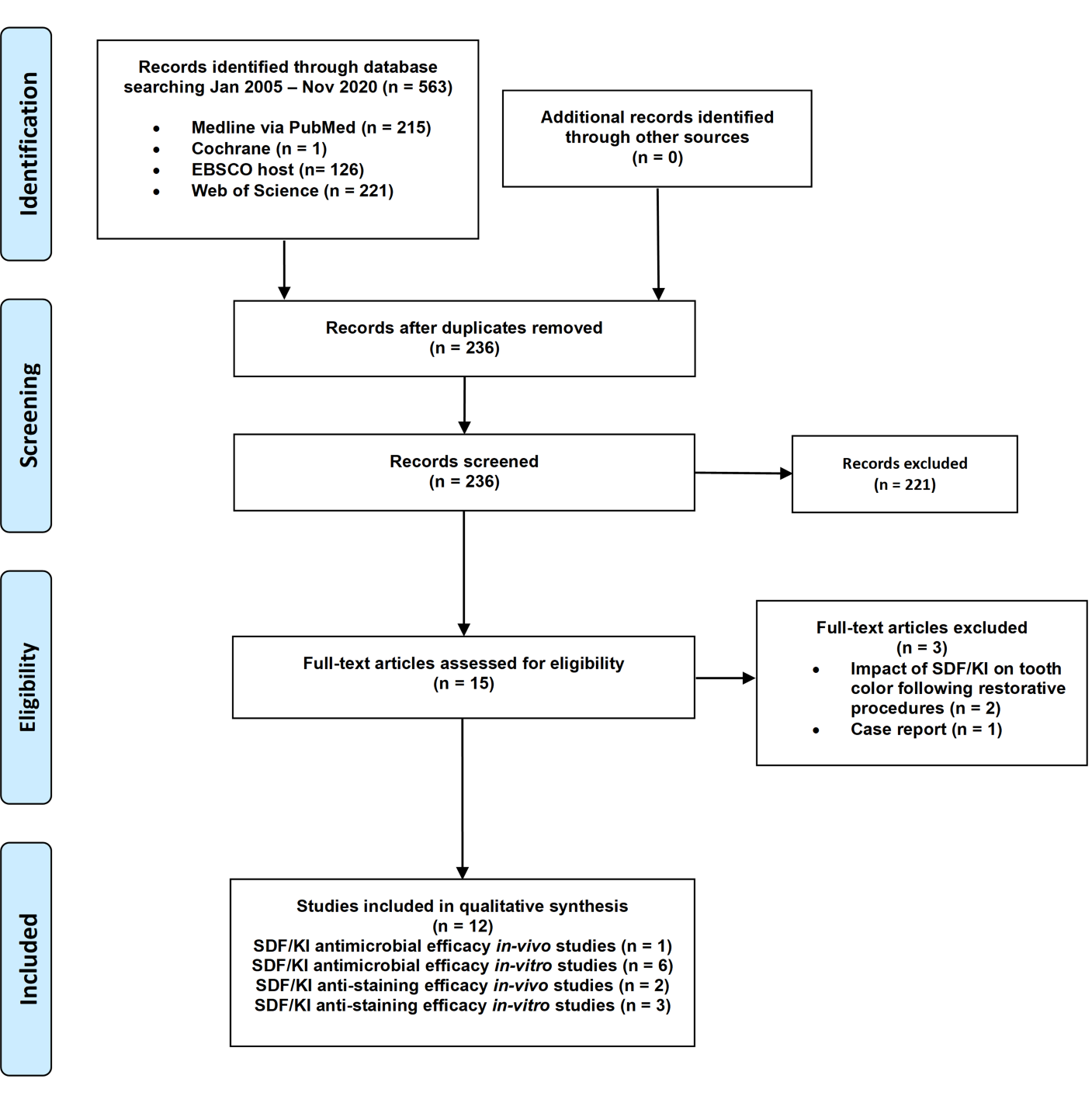

Supplement: S1 Fig — (TIFF) [file pone.0252734.s001.tiff]
